# Personality Heterogeneity Shapes the Neural Organization of Conditioned Threat Learning

**DOI:** 10.21203/rs.3.rs-10020555/v1

**Published:** 2026-07-03

**Authors:** Kai Zhang, Zhenfu Wen, Lihan Cui, Zakiya Atiyah, Isabel Moallem, Hani Meelad, Borrelli Joe, Isabella Mason, Mohammed R. Milad

**Affiliations:** a. Faillace Department of Psychiatry and Behavioral Sciences, McGovern Medical School, University of Texas Health Science Center at Houston, Houston, Texas, USA

## Abstract

How individual differences in personality structure relate to the neural mechanisms of fear conditioning remains an important question for precision psychiatry. Here, combining multidimensional personality profiling with spatiotemporal neuroimaging analyses in a multi-site cohort, we show that personality-defined heterogeneity is associated with distinct neural organization of conditioned threat learning. Rather than reflecting exaggerated neural responses to threat, elevated neuroticism was characterized by a reduced capacity to differentiate conditioned threat from safety signals. This pattern was reflected in reduced threat-related responses within a distributed set of regions, including the ventromedial prefrontal cortex (vmPFC), subgenual anterior cingulate cortex (sgACC), and posterior cingulate cortex (PCC). Complementing the brain activation findings, personality-related differences were also observed in threat–safety dynamic functional connectivity differentiation, including connections between the PCC and centromedial amygdala (cmAMY), precentral cortex and middle frontal gyrus (MFG), and dorsal anterior cingulate cortex (dACC) and orbitofrontal cortex (OFC), as well as broader interactions involving the dorsal attention network (DAN), cerebellum network (CEM) frontoparietal control network (CON), and sensorimotor network (SMN). Symptom analyses revealed that the high-neuroticism subgroup showed elevated trait-related anxiety measures, whereas state anxiety and subjective fear-learning behavior did not differ between groups. Cross-validated predictive analyses further showed that fear-learning-related neural features significantly predicted anxiety sensitivity only within the high-neuroticism subgroup, suggesting personality-dependent brain–symptom coupling. These findings indicate that personality-defined heterogeneity is associated with both the neural organization of conditioned threat learning and the mapping between neural threat representations and anxiety vulnerability, highlighting the importance of incorporating personality structure into neurobiological models of anxiety vulnerability and precision psychiatry frameworks.

## Introduction

Humans differ remarkably in how they perceive, learn, and adapt to threat in an everchanging environment^1,2^. It is widely recognized that individuals with heightened emotional and affective sensitivity are substantially more vulnerable to developing stress-related conditions and severe clinical anxiety^3–6^. Crucially, the psychopathological core of both stress- and anxiety-related conditions is increasingly understood as a fundamental dysregulation in how the nervous system monitors threats and computes safety inputs^7–11^. This universal observation has led to the prevailing assumption that stable, lifelong personality structures fundamentally shape the neurobiological mechanisms through which threat-related information is perceived and regulated^12–14^. Among the established frameworks of human personality, Neuroticism—the dispositional tendency to experience negative affect and perceive the world as threatening—stands as the single most robust predictor of individual psychiatric vulnerability^15,16^. Yet, despite decades of psychiatry confirmation linking high affective sensitivity to clinical symptom development^17–20^, the foundational question remains unresolved: how distinct personality profiles differentially shape the neural representations of conditioned threat learning.

Pavlovian fear conditioning provides a powerful, highly translational experimental framework for investigating how the human brain acquires, represents, and regulates learned threat associations^21–24^. By pairing previously neutral stimuli (CS+) with an aversive outcome while maintaining an unreinforced safety stimulus (CS-), this paradigm enables the precise quantification of associative learning across neural, behavioral, and physiological levels. Traditional neuroimaging studies mapped personality-dependent vulnerabilities by focusing on the response magnitude of neural activation, operationalizing affective sensitivity as an exaggerated functional reactivity to danger^25,26^. However, empirical findings built upon this assumption remain controversial and contradictory^27,28^. One possible explanation is that affective sensitivity may not be adequately characterized by threat reactivity alone. In adaptive fear learning, successful regulation depends not only on detecting threat, but also on accurately distinguishing threat from safety signals. This raises a critical question for psychiatric neuroscience: do personality-related differences in affective sensitivity arise primarily from variations in threat responsiveness, or from differences in how the brain represents and differentiates threat and safety information?

A further question is whether personality heterogeneity also shapes the relationship between neural mechanisms of threat learning and anxiety-related symptom expression. Recent advances in affective neuroscience and precision psychiatry have increasingly focused on identifying neural markers that can explain individual differences in behavioral adaptation and clinical outcome^29–31^. Such markers are valuable not only for clarifying the neurobiological basis of human behavior^32,33^, but also for informing clinically relevant stratification, prognosis, and intervention strategies^34–38^. However, the explanatory and predictive utility of neural markers may depend on latent individual differences. Personality structure represents a particularly important source of such heterogeneity because it influences both emotional processing and vulnerability to psychopathology. In this context, it remains unclear whether neural signatures of fear learning relate to anxiety-related vulnerability uniformly across individuals, or whether this brain–symptom relationship depends on personality-defined heterogeneity. It is also unknown which neural features most strongly support such associations—regional activation or dynamic functional connectivity—and whether the relevant symptom dimensions reflect stable trait vulnerability or transient state anxiety. Addressing these questions may help clarify how personality structure links threat-learning neurobiology to anxiety-related vulnerability.

To address these fundamental questions, the present study leveraged a multi-site cohort of 249 participants who completed a Pavlovian fear conditioning paradigm during functional MRI acquisition, alongside comprehensive personality and anxiety-related symptom assessments. A central methodological challenge was how to define personality-related heterogeneity in a way that preserved the multidimensional nature of personality. Traditional personality neuroscience frequently relies on single-trait correlations or arbitrary score-splitting (such as median-splits), which ignore individual variability, and isolate single personality dimensions from their broader psychological context^39,40^. To overcome these limitations, we adopted a data-driven, unsupervised Gaussian mixture modeling approach using all five dimensions of the NEO Five-Factor Inventory-3. By treating personality profiles as multidimensional configurations rather than isolated trait scores, this approach identified distinct latent personality subgroups, primarily characterized by divergent neuroticism profiles. We then examined whether latent personality heterogeneity is associated with distinct neural organization of threat learning. By integrating neural decoding, task-evoked activation, dynamic functional connectivity, behavioral fear responses, symptom profiling, and brain–symptom predictive modeling, we tested three related questions: whether personality-defined subgroups differ in their neural representation of conditioned threat and safety signals, whether such differences are reflected in behavioral and symptom-level vulnerability, and whether the relationship between neural threat representations and anxiety-related symptoms is itself shaped by personality structure.

## Result

A central question of this study was whether individual differences in personality structure are associated with how the human brain acquires conditioned threat and processes safety signals. To address this, we leveraged data-driven, unsupervised Gaussian Mixture Modeling (GMM) of five-factor personality profiles to stratify participants into distinct latent subgroups, which were predominantly characterized by diverging levels of neuroticism (i.e., low-neuroticism and high-neuroticism groups). We subsequently evaluated whether these two personality-defined subgroups exhibited differences across distributed neural representations, regional activation, dynamic functional connectivity, and anxiety-related symptom profiles. Across multiple levels of neural measures, the low-neuroticism subgroup demonstrated greater neural differentiation between threat and safety signals during early fear learning compared to the high-neuroticism subgroup. First, neural decoding analyses revealed that while both subgroups exhibited comparable baseline fear probability expressions to safety cues (CS-), the low-neuroticism subgroup showed significantly higher fear probability expression specifically in response to conditioned threat cues (CS+) during the early acquisition phase. Second, univariate activation analyses of the early conditioning contrast (CS+ > CS−) revealed greater conditioning-related activation in the low-neuroticism subgroup than in the high-neuroticism subgroup, with significant group differences observed in the ventromedial prefrontal cortex (vmPFC), subgenual anterior cingulate cortex (sgACC), and posterior cingulate cortex (PCC) after correction for multiple comparisons. Third, task-based dynamic functional connectivity analyses also demonstrated greater threat–safety connectivity differentiation (CS+ > CS-) in the low-neuroticism subgroup, spanning both the 24-node threat-circuit atlas and large-scale whole-brain network interactions. At the symptom level, the low-neuroticism subgroup exhibited lower anxiety-related symptom scores than the high-neuroticism subgroup. These differences were primarily observed for trait-related vulnerability measures, including ASI-3, ASI-Total, and STAI-Trait, whereas no significant subgroup difference was observed for state anxiety (STAI-State). Finally, multivariate pattern predictive modeling further revealed subgroup-specific brain–symptom associations: fear-learning-related neural features did not significantly predict symptom severity in the low-neuroticism subgroup, but significant cross-validated predictions emerged selectively within the high-neuroticism subgroup. These findings indicate that low-neuroticism individuals exhibit stronger neural differentiation between threat and safety signals during early fear learning, whereas high-neuroticism individuals show weaker threat-safety neural differentiation and stronger coupling between fear-learning neural representations and anxiety-related symptom severity.

### Personality-based Clustering Identifies Distinct Low- and High-Neuroticism Subgroups

**Figure 1.**
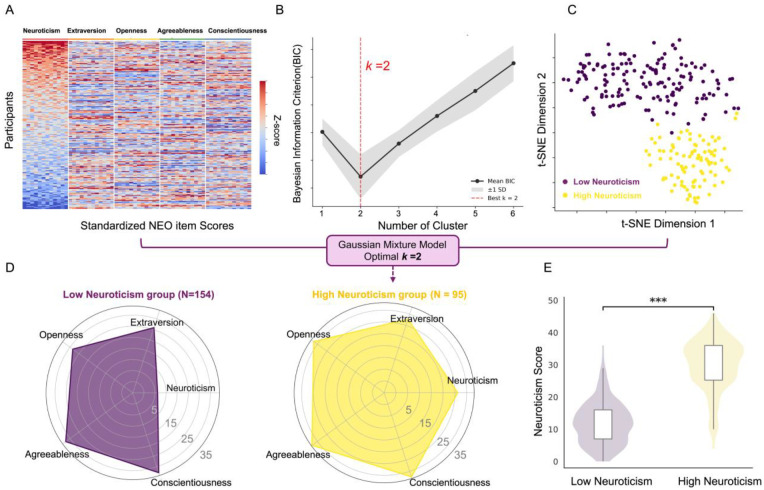
Data-driven personality clustering identifies distinct neuroticism-defined subgroups. A. Heatmap visualization of standardized responses across all 60 NEO-FFI-3 items for all participants. B. Bayesian Information Criterion (BIC) model selection results across different cluster numbers, identifying the two-cluster solution (*k* = 2) as the optimal model. Shaded regions indicate ±1 standard deviation across subsampling iterations. C. t-distributed stochastic neighbor embedding (t-SNE) visualization of subgroup separation based on NEO personality profiles. D. Radar plots illustrating the mean personality profiles of the identified subgroups across the five NEO personality dimensions. One subgroup was characterized by relatively lower neuroticism scores (low-neuroticism group), whereas the second subgroup exhibited markedly elevated neuroticism scores (high-neuroticism group). E. Group comparison of neuroticism scores between the identified subgroups. The high-neuroticism subgroup exhibited significantly greater neuroticism levels compared with the low-neuroticism subgroup (p < 0.001).

Using standardized responses from all 60 items of the NEO Five-Factor Inventory (NEO-FFI-3) as input features (Figure 1A), unsupervised Gaussian mixture model (GMM)-based clustering stratified participants into two latent personality-defined subgroups. Bayesian Information Criterion (BIC)-based model selection consistently identified a two-cluster solution as the optimal model (Figure 1B), and subgroup separation was further visualized using t-distributed stochastic neighbor embedding (t-SNE), demonstrating clear segregation between the identified personality profiles (Figure 1C). Although all five personality dimensions contributed to the clustering procedure, characterization of the resulting subgroups revealed that neuroticism represented the principal dimension differentiating the two profiles (Figure 1D). One subgroup exhibited significantly lower neuroticism scores and was therefore designated the low-neuroticism subgroup (n = 154), whereas the second subgroup exhibited significantly higher neuroticism scores (p < 0.001; Figure 1E). and was designated the high-neuroticism subgroup (n = 95). Differences across the remaining personality dimensions are summarized in Supplementary Table 1 (Supplement Material).

To evaluate the robustness of the personality stratification, we performed a series of complementary stability analyses. Subsampling-based validation demonstrated that the two-cluster solution was consistently favored across repeated random subsets of participants. Across 50 independent subsampling iterations, the Bayesian Information Criterion (BIC) reached its minimum at *k* = 2 and remained stable across resampled datasets (Figure 1B). Additional analyses further supported the reproducibility of the clustering solution. Repeated model fitting across independent random initializations yielded highly consistent subtype assignments, as reflected by high agreement metrics based on the Adjusted Rand Index (ARI) and Normalized Mutual Information (NMI). Similarly, split-half reproducibility analyses demonstrated strong concordance between subtype assignments derived from independent training and testing subsets (See Supplement Material for more detailed results).

### Decoding Performance for Fear Conditioning Between Two Subgroups

**Figure 2.**
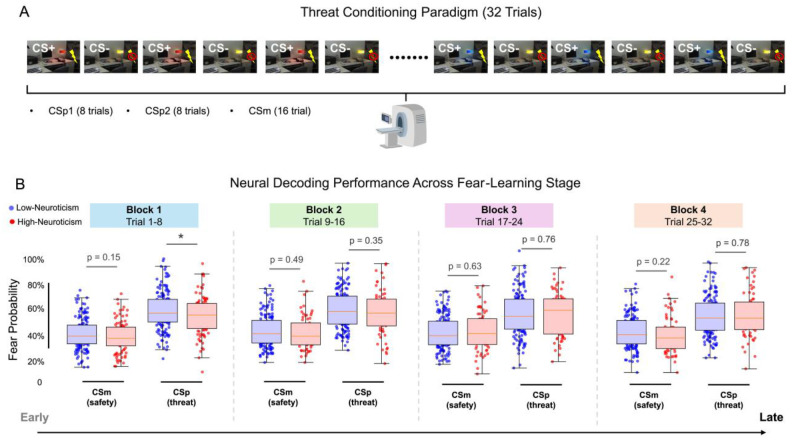
Neural decoding performance during fear conditioning. A. Schematic illustration of the Pavlovian fear conditioning paradigm. B. Neural decoding performance across four successive conditioning blocks for the two personality-defined subgroups during fear learning. (Throughout the manuscript, conditioned threat cues (CS+) correspond to the conditioned stimulus paired with shock (CSp), whereas conditioned safety cues (CS−) correspond to the unpaired conditioned stimulus (CSm). The terms CS+/CSp and CS−/CSm are therefore used interchangeably.)

To characterize personality-related differences in conditioned threat representations, we applied a previously validated distributed neural threat decoder^41^ across the fear conditioning phase. Decoder analyses were performed separately for conditioned threat (CS+) and safety (CS−) cues across four successive conditioning blocks, each consisting of four trials. Decoder inputs consisted of block-averaged GLM activation maps derived from the 24-node threat-circuit atlas, and decoder outputs were expressed as fear probability scores ranging from 0 to 1, with higher values indicating stronger conditioned threat representation. A repeated-measures generalized estimating equation (GEE) model was used to evaluate decoder-derived fear probability across conditioning conditions, blocks, and personality-defined subgroups. Across conditioning blocks, CS− cues consistently elicited low fear probability values and showed no significant subgroup differences following correction for multiple comparisons (Figure 2B). In contrast, subgroup differences emerged selectively during conditioned threat processing. The low-neuroticism subgroup exhibited higher fear probability expression to CS+ cues across the early conditioning phase, with the largest effect observed during the first conditioning block. Post hoc comparisons revealed that this early-stage subgroup difference remained significant following false discovery rate (p_FDR<0.05) correction, whereas subsequent blocks showed non-significant differences. Within-group comparisons further confirmed successful neural threat–safety discrimination (CS+ > CS−) across all conditioning blocks in both personality-defined subgroups (all p_FDR < 0.05). The magnitude of this threat–safety differentiation was greatest during the first conditioning block, where the low-neuroticism subgroup exhibited a substantially larger threat–safety contrast than the high-neuroticism subgroup (Low-Neuroticism: t = 15.89, p_FDR = 2.18e-33; High-Neuroticism: t = 8.59, p_FDR = 3.10e-13). These results indicate that individuals with lower neuroticism exhibit stronger neural differentiation between conditioned threat and safety cues during early fear learning, whereas individuals with higher neuroticism show reduced threat–safety differentiation, primarily driven by weaker neural expression of conditioned threat cues during acquisition.

### Distinct Neural Organization of Fear Learning Across Personality-Defined Subgroups

Building on the neural decoding results, we next examined whether personality-defined subgroups also differed in the neural mechanisms supporting early fear learning. Consistent with the decoder findings, activation analyses of the early conditioning contrast (CSp−CSm) revealed stronger fear-learning-related neural responses in the low-neuroticism subgroup compared with the high-neuroticism subgroup. This effect was observed within key threat-regulatory regions, including the PCC, vmPFC, and sgACC, surviving family-wise error correction (p < 0.05). Similar spatial patterns were also observed in the whole-brain mask analysis, indicating convergent evidence across complementary ROI-level and whole-brain parcellation schemes.

Dynamic functional connectivity estimated using a jackknife correlation procedure further supported this pattern. We specifically quantified threat–safety connectivity differentiation using a connectivity contrast metric (ΔFC = FC_CSp – FC_CSm), defined as the difference between conditioned threat and safety cue connectivity during the block 1. Within the 24-node threat-circuit atlas, the low-neuroticism subgroup exhibited greater threat–safety connectivity differentiation than the high-neuroticism subgroup, with significant effects observed for the PCC–cmAMY, dACC–OFC, and precentral–MFG connections (pFDR < 0.05). At the network level (within whole brain mask), whole-brain connectivity analyses revealed stronger subgroup differences involving DAN (dorsal attention network)–CEM (cerebellum network), CEM–CEM, CON (frontoparietal control network)–CEM, and DAN–SMN (sensorimotor network) network interactions (p_FDR < 0.05).

### Personality-Defined Subgroups Exhibit Distinct Trait-Related Anxiety Profiles

**Figure 4.**
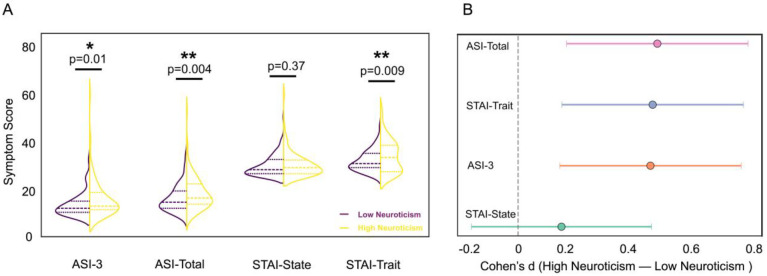
Personality-defined subgroup differences in anxiety-related symptom profiles. A. Group comparisons of anxiety-related symptom measures between low- and high-neuroticism subgroups. The high-neuroticism subgroup exhibited significantly elevated trait-related symptom measures compared with the low-neuroticism subgroup. B. Effect size analysis of subgroup differences across symptom measures.

Having identified personality-dependent differences in neural threat learning, we next examined whether these subgroups also differed in anxiety-related symptom profiles. Consistent with this broader pattern of subgroup divergence, the high-neuroticism subgroup exhibited significantly elevated anxiety-related symptom measures compared with the low-neuroticism subgroup (Figure 4A). Importantly, subgroup differences were most prominent for trait-related vulnerability measures rather than transient state-dependent anxiety responses. Specifically, the high-neuroticism subgroup showed significantly higher scores on ASI-3, ASI-Total, and STAI-Trait measures, whereas no significant subgroup difference was observed for STAI-State scores. These findings suggest that the identified personality-defined subgroups primarily differ in stable affective vulnerability traits rather than momentary emotional state fluctuations. Effect size analyses further supported this pattern (Figure 4B). Cohen’s d values demonstrated moderate subgroup effects for ASI-3 (Cohen's d = 0.38, 95% CI = 0.12–0.64, p = 0.01), ASI-Total (Cohen's d = 0.40, 95% CI = 0.14–0.66, p = 0.004), and STAI-Trait (Cohen's d = 0.39, 95% CI = 0.12–0.65, p = 0.009) measures, whereas STAI-State (Cohen's d = 0.12, 95% CI = −0.13–0.38, p = 0.37) exhibited minimal effect size differences between subgroups. These findings indicate that individuals with high neuroticism exhibit elevated anxiety sensitivity and trait anxiety, whereas transient state anxiety does not differ substantially between personality-defined subgroups.

### Brain–Symptom Predictive Correlation Between Subgroups

**Figure 5.**
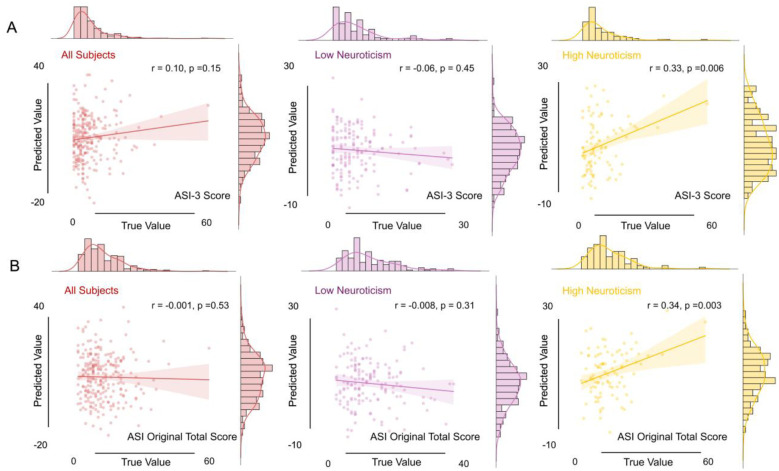
Personality-dependent prediction of anxiety-related symptoms from fear-learning neural representations. A. Cross-validated prediction of ASI-3 scores using fear-learning-related neural features derived from conditioning contrasts (CSp−CSm). Significant brain–symptom prediction was observed selectively in the high-neuroticism subgroup. B. Cross-validated prediction of ASI-Total scores using fear-learning-related neural features derived from conditioning contrasts (CSp−CSm). Significant predictive associations were observed only in the high-neuroticism subgroup, whereas no significant prediction was detected in the low-neuroticism subgroup or the full sample. Shaded regions indicate confidence intervals around regression fits.

Given the observed subgroup differences in fear-learning-related neural organization and anxiety-related symptom profiles, we next examined whether neural representations of conditioned threat learning were differentially associated with symptom severity across personality-defined subgroups. To address this question, neural activation features derived from the threat–safety contrast (CSp−CSm) were used to predict individual anxiety-related symptom measures using cross-validated linear regression models. At the whole-sample level, fear-learning-related activation features did not significantly predict either ASI-3 or ASI-Total symptom scores (Figure 5). Similarly, no significant brain–symptom predictive relationship was observed within the low-neuroticism subgroup. In contrast, significant and cross-validated predictive associations emerged selectively within the high-neuroticism subgroup. Specifically, fear-learning-related activation patterns significantly predicted both ASI-3 (r = 0.33, p = 4 × 10^−4^, MSE = 87, permutation p = 0.007) and ASI-Total scores (r = 0.34, p = 6 × 10^−4^, MSE = 67, permutation p = 0.004). Permutation distributions supporting model significance are provided in Supplementary Figure S1. In both cases, stronger threat–safety activation contrasts were associated with greater anxiety-related symptom severity. These findings indicate that brain–symptom associations were selectively detectable within the high-neuroticism subgroup. Comparable predictive relationships were not observed in the low-neuroticism subgroup, and connectivity-based models did not yield significant prediction performance in either subgroup.

### Behavioral Performance Across Personality-Defined Subgroups

To determine whether personality-defined subgroup differences were also evident at the behavioral level, we compared subjective fear-learning responses (quantified as the change in self-reported fear ratings—post-conditioning minus pre-conditioning) between personality-defined subgroups. Across all participants, conditioned threat cues elicited greater increases in fear ratings than safety cues, confirming successful fear acquisition at the behavioral level (Supplementary Figure S2). However, despite pronounced subgroup differences in neural representations of fear learning and anxiety-related symptom profiles, no significant differences in conditioned fear ratings were observed between the low- and high-neuroticism subgroups. Both groups exhibited comparable behavioral evidence of fear learning. These findings suggest that personality-dependent differences in fear learning are more readily detectable at the neural and trait-related symptom levels than in overt behavioral responses.

## Discussion

The inherent heterogeneity in human personality architecture, particularly characterized by emotional sensitivity profiles, systematically shapes the brain capacity to process and learn from conditioning threat. By combining multi-dimensional personality profiling with comprehensive neural trait profiling, we demonstrate that individual differences in personality do not act as a simple “volume control” for threat response, but rather serve as an “emotion filter” through which the human brain precisely discriminates threat from safety. This neural divergence is anatomically anchored within the distinct regions comprising the vmPFC, sgACC, and PCC. Further, we show that this representation can predict trait-based anxiety in high-neuroticism individual rather than low-neuroticism individual. All these findings not only advance our mechanistic understanding of how human personality shapes the processing of conditioned threat, but also provide a potential neurobiological strategy for implementing stratified, personalized interventions in precision psychiatry.

Neural decoding and activation analyses converged on a common finding: high-neuroticism individuals exhibited weaker neural differentiation between conditioned threat and safety cues during early fear learning. Neural decoding revealed lower fear-probability expression in response to conditioned threat, while activation analyses localized this effect to reduced threat-related responses within the vmPFC, sgACC, and PCC. Notably, neural responses to safety cues remained largely comparable across subgroups, indicating that the observed group differences were primarily driven by attenuated processing of conditioned threat signals. As a result, high-neuroticism individuals exhibited a less differentiated neural representation of danger relative to safety during threat acquisition. Neuroanatomically, the vmPFC and sgACC are thought to contribute to the representation of the predictive and affective significance of conditioned stimuli. The reduced responses observed in these regions suggest that conditioned threat cues may engage these evaluative processes to a lesser extent in high-neuroticism individuals during fear learning^9,42,43^. A similar pattern was observed in the PCC, a core hub of the default mode network implicated in contextual evaluation and internally directed processing^44^. Reduced PCC responses to conditioned threat cues may be consistent with less prominent incorporation of threat-related information into broader contextual representations during fear learning. Collectively, these findings suggest that reduced neural differentiation between threat and safety cues represents a key neurofunctional characteristic associated with neuroticism-related variation in conditioned threat learning.

Complementing the activation and decoding findings, dynamic functional connectivity (dFC) analyses further showed that personality-defined differences in fear learning were expressed at the level of trial-wise network coordination. Within the 24-node threat-circuit atlas, the high-neuroticism subgroup showed weaker fear-learning-related connectivity than the low-neuroticism subgroup for several connections during the CS+ > CS− contrast, including PCC–cmAMY, precentral–MFG, and dACC–OFC. The reduced PCC–cmAMY coupling suggests weaker functional coordination between contextual-evaluative regions and amygdala-centered threat-processing circuitry during conditioned threat learning in high-neuroticism individuals^45,46^. Similarly, weaker dACC–OFC and precentral–MFG connectivity may reflect reduced coordination among regions involved in threat-value evaluation, action preparation^47–49^, and executive control during early threat acquisition. Whole-brain network-level analyses showed a convergent pattern, with group differences concentrated across connections involving the DAN, CEM, CON, and SMN networks. These effects suggest that high-neuroticism individuals exhibited less pronounced fear-learning-related coordination among attention, core integration, control, and sensorimotor systems. Viewed together, the activation and dFC findings suggest that reduced neural differentiation between threat and safety cues in high-neuroticism individuals is expressed not only at the level of regional responses, but also in the dynamic coordination of distributed threat-processing networks.

Our symptom analyses revealed a striking dissociation between trait- and state-related measures. Compared with the low-neuroticism subgroup, high-neuroticism individuals exhibited significantly elevated scores on trait-oriented anxiety measures, including STAI-T, ASI-Total, and ASI-3, whereas no significant difference was observed for state anxiety (STAI-S). Notably, this symptom profile closely paralleled the neural findings. The reduced threat-related activation and weaker fear-learning-related connectivity observed in the high-neuroticism subgroup were expressed during a stable conditioning paradigm and therefore likely reflect enduring differences in threat-processing organization rather than transient fluctuations in emotional state. Consistent with this interpretation, subjective fear ratings acquired during the task did not differ between groups despite pronounced differences in neural responses and trait symptom measures. Together, these findings suggest that personality-dependent differences in fear learning are more strongly associated with stable vulnerability-related traits than with momentary emotional states.

This interpretation is further supported by the brain–symptom prediction analyses. Fear-learning-related neural activation significantly predicted ASI-3 and ASI-Total scores exclusively within the high-neuroticism subgroup, whereas no significant prediction was observed in the low-neuroticism subgroup or the combined cohort. These findings suggest that individual differences in threat-related neural representations are more closely associated with anxiety-related symptom expression among highly neurotic individuals. In contrast, the absence of significant prediction in the low-neuroticism subgroup indicates that variability in symptom scores may be less dependent on fear-learning-related neural processes within this population. From a precision psychiatry perspective, these results further suggest that personality structure may shape the strength and detectability of brain–symptom relationships. Consequently, accounting for personality-defined heterogeneity may improve the identification of biologically meaningful subgroups and enhance the interpretability of brain-based markers of psychiatric vulnerability.

Beyond its implications for understanding personality-dependent fear learning, the present findings may also inform future efforts in precision psychiatry. A growing body of work has proposed that neural signatures of threat processing may serve as biologically meaningful markers for characterizing anxiety vulnerability, predicting treatment response, and guiding personalized interventions^50–52^. Our findings extend this framework by suggesting that the clinical utility of such threat-related neural markers may depend, in part, on underlying personality heterogeneity. Specifically, personality-defined subgroups differed not only in neural threat processing and symptom burden, but also in the strength of brain–symptom coupling. These findings suggest that incorporating multidimensional personality profiling into baseline clinical assessment may improve biologically informed patient stratification and facilitate the development of more personalized intervention strategies tailored to distinct neurobehavioral profiles. The present findings may also provide candidate neural targets for future neuromodulation-based interventions. In particular, the vmPFC, sgACC, and PCC emerged as convergent regions associated with personality-dependent differences in threat learning and anxiety-related vulnerability. Although the current study cannot establish causal relationships, these regions may represent promising targets for future causal manipulation studies using transcranial magnetic stimulation^53^ (TMS), transcranial direct current stimulation^54^ (tDCS), or real-time fMRI neurofeedback^55^. Future work may determine whether modulating activity within these circuits, or altering their associated network dynamics, can shift threat-processing representations toward more adaptive configurations and reduce anxiety-related vulnerability in highly neurotic individuals. Such approaches could provide an important bridge between computational models of personality heterogeneity and individualized neuropsychiatric interventions.

Several limitations should be considered when interpreting the present findings. First, although our study identified robust personality-dependent differences in fear-learning-related neural representations, symptom profiles, and brain–symptom coupling, all analyses were conducted in a non-clinical population. Due to the lack of an independent psychiatric dataset containing comparable personality, neuroimaging, and fear-learning measures, we were unable to directly evaluate the generalizability of these findings to clinical populations. Future studies should examine whether similar personality-defined neural subtypes can be identified in individuals with anxiety-related disorders and whether these subgroup-specific neural signatures are associated with symptom severity, treatment response, or disease progression. Second, the current study focused exclusively on the acquisition phase of conditioned threat learning. While fear conditioning provides a critical framework for understanding how threat associations are formed, the ability to extinguish previously learned fear responses is equally important for adaptive emotional regulation and is highly relevant to anxiety psychopathology. It therefore remains unclear whether personality-defined heterogeneity similarly influences the neural organization of fear extinction learning. Future work extending this framework to extinction and extinction-recall processes may provide a more comprehensive understanding of how personality shapes the full trajectory of adaptive and maladaptive threat regulation.

## Method

### Participants

A total of 249 participants (mean age = 28.8 ± 10.6 years, range = 18–60 years; 96 males, 152 females, and 1 participant reporting another gender) were recruited across three independent acquisition sites: New York University Grossman School of Medicine (Site 1, n = 76) and two imaging cohorts at The University of Texas Health Science Center at Houston (UTHealth Houston), which were treated as separate acquisition sites due to differences in MRI scanner platforms and imaging protocols (Site 2, n = 138; Site 3, n = 35). Exclusion criteria included a history of major neurological disorders, severe psychiatric illness, significant medical conditions affecting central nervous system function, and standard contraindications to MRI scanning. All participants provided written informed consent in accordance with the Declaration of Helsinki. Study procedures were approved by the Institutional Review Boards of the participating institutions.

### Fear Conditioning Paradigm

Participants completed a Pavlovian fear conditioning and extinction paradigm from our previously established protocol^56,57^ (Figure 2A). Because the present study specifically focused on conditioned threat learning, only data acquired during the fear conditioning phase on Day 1(Conditioning phase) were included in the current analyses. Conditioned threat learning was induced using visual conditioned stimuli (CS) paired with aversive electric shocks delivered through a BIOPAC MP160 system (BIOPAC Systems Inc., Goleta, CA, USA). During the conditioning phase, participants viewed visual stimuli consisting of three colored cues. Two colors served as conditioned threat cues (CS+1 and CS+2, reinforcement rate: 62.5%), whereas a third color served as a safety cue (CS−). Across the experiment, a total of 32 trials were presented, including 8 CS+1 trials, 8 CS+2 trials, and 16 CS− trials. Each trial began with the presentation of a contextual background image (office scene) for 3 s, followed by the conditioned stimulus for 6 s. For CS+ trials, a 500 ms electric shock was delivered at stimulus offset, whereas CS− trials were never reinforced. The inter-trial interval (ITI) was jittered between 12, 15, and 18 s (mean = 15 s). Shock intensity was individually calibrated prior to scanning to be highly aversive but not painful. To minimize potential confounding effects related to perceptual properties of specific stimulus colors, the assignment of colors to CS+ and CS− conditions was counterbalanced across participants using eight randomized experimental versions.

### Experimental Measures

All participants underwent multimodal assessment including structural and functional neuroimaging, personality assessment, anxiety-related symptom evaluation, and fear-learning behavioral measurements. MRI acquisition included high-resolution T1-weighted structural imaging and blood-oxygen-level-dependent (BOLD) functional MRI collected during the Day 1 fear conditioning phase. Personality traits were assessed prior to the experiment using the NEO Five-Factor Inventory-3 (NEO-FFI-3), a 60-item self-report questionnaire measuring Neuroticism, Extraversion, Openness, Agreeableness, and Conscientiousness^58,59^. Baseline anxiety-related measures included the State-Trait Anxiety Inventory Form Y-1 (STAI-Y1; state anxiety), State-Trait Anxiety Inventory Form Y-2 (STAI-Y2; trait anxiety)^60^, Anxiety Sensitivity Index-3 Total Score (ASI-3 Total)^61^, and the original Anxiety Sensitivity Index total score (ASI Total)^62^. Behavioral fear-learning performance was assessed using subjective fear ratings collected before and after fear conditioning. Fear-learning behavioral indices were quantified as the change in subjective fear ratings from pre-conditioning to post-conditioning (post-learning minus pre-learning). NEO-FFI-3 measures were used for personality-based clustering analyses, whereas symptom and behavioral measures were used for subgroup comparisons and brain–behavior association analyses.

### Neuroimaging Acquisition

For stie 1 and site 2, Neuroimaging data were collected on a data were acquired on a 3T Siemens Prisma using a 64-channel head coil. High-resolution T1-weighted anatomical images were acquired for spatial normalization. functional images were collected with a gradient-echo echo-planar imaging (EPI) sequence (TR = 1500 ms; TE = 35 ms; flip angle = 62°; voxel size = 2 × 2 × 2 mm; FOV = 208 mm; 72 slices, interleaved, multi-band acceleration factor = 4).

For site 3, Neuroimaging data will be collected on a 3T Siemens MAGNETOM using a 64-channel head coil. Functional images will be collected with a gradient-echo echo-planar imaging (EPI) sequence (TR = 705 ms; TE = 30 ms; flip angle = 52°; voxel size = 2 × 2 × 2 mm; FOV = 208 mm; 72 slices, interleaved, multi-band acceleration factor = 8).

Data preprocessing was performed using fMRIPrep version 20.0.6^63^. T1-weighted images underwent intensity nonuniformity correction (N4BiasFieldCorrection, ANTs 2.3.3), skull stripping, tissue segmentation (gray matter, white matter, CSF), and nonlinear normalization to MNI152NLin2009cAsym space (ANTs’ antsRegistration). Functional images were corrected for head motion with mcflirt (FSL), adjusted for slice-timing using 3dTshift (AFNI), and co-registered to the corresponding T1-weighted image using boundary-based registration using flirt (FSL). The resulting functional data were normalized to standard MNI space and resampled to 2 mm isotropic resolution using Lanczos interpolation.

### Gaussian Mixture Model-based Personality Stratification

Participants were stratified into personality-defined subgroups using an unsupervised Gaussian Mixture Model (GMM) based on the five-factor personality dimensions derived from the NEO inventory, including Neuroticism, Extraversion, Openness, Agreeableness, and Conscientiousness. Prior to clustering, all personality variables were z-score standardized across participants to ensure comparable scaling across dimensions. GMMs with varying numbers of latent clusters (k = 1–6) were estimated using full covariance matrices and maximum-likelihood optimization. To reduce sensitivity to local minima, each model was initialized with 20 independent random starts. Model selection was performed using the Bayesian Information Criterion (BIC), with the optimal number of clusters defined as the solution minimizing the BIC across candidate models. Following model selection, each participant was assigned to the subtype with the highest posterior probability under the fitted GMM. Compared with distance-based clustering approaches, GMM provides a probabilistic framework capable of modeling heterogeneous covariance structures and estimating soft cluster membership, thereby offering improved sensitivity for capturing latent personality-defined population structure.

### Cluster Stability and Reproducibility Analysis

To evaluate the robustness and reproducibility of the identified personality subtypes, we performed three complementary stability analyses designed to test whether the clustering solution remained stable under variations in model initialization, sample composition, and out-of-sample generalization conditions. First, we conducted a subsampling-based stability analysis. Across 50 independent iterations, 80% of participants were randomly sampled without replacement, and GMMs were re-estimated across candidate cluster solutions (k = 1–6). For each iteration, Bayesian Information Criterion (BIC) values were computed to assess the consistency of model selection across subsamples. Second, we assessed random-seed stability of the clustering solution. The optimal GMM model was repeatedly fitted using 100 independent random initializations. Cluster assignments obtained across runs were compared pairwise using the Adjusted Rand Index (ARI) and Normalized Mutual Information (NMI), both of which are invariant to label permutation and provide quantitative measures of clustering consistency across stochastic model initializations. Third, we evaluated split-half reproducibility using repeated train-test partitioning. In each repetition, participants were randomly divided into training and testing subsets of equal size. A GMM was fitted exclusively on the training subset and subsequently used to predict subtype assignments in the held-out testing subset. Independently, a separate GMM was fitted directly on the testing subset. Agreement between predicted subtype labels and independently derived subtype labels was quantified using ARI and NMI across 100 repeated random partitions. This procedure assessed the extent to which the identified subtype structure generalized across independent participant subsets and was not driven by sample-specific clustering patterns.

### Brain Parcellation and Regions of Interest

To characterize conditioning-related neural responses at both circuit-level and whole-brain scales, two complementary brain parcellation schemes were used. First, analyses focused on an extended threat-circuit atlas derived from the previously published study, comprising 24 regions of interest associated with threat and safety processing circuitry^41^. In addition, whole-brain analyses were performed using a 442-region functional parcellation atlas consisting of 400 cortical regions^64^, 32 subcortical regions^65^, and 10 cerebellar regions^66^. This whole-brain parcellation framework enabled large-scale characterization of distributed activation and connectivity patterns during fear conditioning.

### Neural Decoding Analysis

Conditioned threat representations were quantified using a previously validated distributed neural threat decoder developed in the previous study^41^. Decoder analyses were performed across the full fear conditioning phase, including all four conditioning blocks. For each participant, whole-brain activation patterns associated with conditioned threat cues (CS+) and safety cues (CS−) were projected onto the pretrained threat decoder to estimate trial-wise conditioned threat expression. Decoder outputs were summarized as fear probability scores ranging from 0 to 1, with higher values indicating stronger expression of conditioned threat-related neural representations. Mean fear probability values for each block were calculated separately for CS+ and CS− conditions across the conditioning phase. Decoding performance was subsequently compared across personality-defined subgroups to characterize individual differences in distributed neural representations of conditioned threat learning.

### Univariate Activation Analysis

Task-related brain activation was estimated using a general linear model (GLM) framework implemented in the Nilearn toolbox. Given the study focus on conditioned threat learning, analyses were restricted to the early fear conditioning phase. Specifically, the first four CS+ trials (CSp01) and the first four CS− trials (CSm01) were modeled separately to capture early-stage acquisition-related neural responses. For each participant, condition-specific regressors were convolved with the canonical hemodynamic response function, and individual-level contrast maps were generated using the contrast CSp01 > CSm01, reflecting differential neural responses to conditioned threat versus safety cues during early fear learning. Group-level analyses subsequently compared these contrast maps across personality-defined subgroups to characterize individual differences in conditioning-related neural activation patterns.

### Dynamic Functional Connectivity Analysis

Task-based dynamic functional connectivity (dFC) was estimated using a jackknife correlation procedure to quantify trial-specific fluctuations in functional coupling during fear learning^67^. Dynamic FC estimation combined the beta-series correlation framework with leave-one-trial-out jackknife correlation analysis. Specifically, trial-wise activation estimates from all conditioning trials were combined, and each trial was iteratively omitted while ROI–ROI correlations were computed from the remaining trials. This procedure generated a trial-specific connectivity estimate reflecting the relative contribution of each trial to the overall connectivity pattern across the conditioning phase. Correlation matrices were subsequently Fisher-z transformed and standardized across trials, resulting in a symmetric trial-wise connectivity matrix for each participant. Dynamic FC analyses were performed using both the 442-region whole-brain atlas and the 24-region threat-circuit atlas, yielding 442 × 442 and 24 × 24 connectivity matrices for each trial, respectively. Connectivity matrices were then separated according to conditioned stimulus type (CS+ and CS−) and ordered by trial presentation sequence. To characterize early-stage fear acquisition, analyses focused on the first conditioning block, corresponding to the initial four CS+ and four CS− trials. For each condition, the magnitude of trial-specific connectivity fluctuations was quantified by averaging jackknife-derived connectivity estimates across trials within the block. Fear-learning-related connectivity differentiation was then calculated as the contrast between conditioned threat and safety trials (ΔFC = FC_CSp − FC_CSm). Group-level analyses subsequently compared these threat–safety connectivity differentiation patterns across personality-defined subgroups.

### Brain–Symptom Predictive Modeling

To examine whether conditioning-related neural features predicted individual differences in anxiety-related symptoms, predictive modeling analyses were performed using cross-validated linear regression. Neural features derived from fear-learning-related activation or connectivity measures were used as predictor variables (X), whereas symptom measures served as target variables (Y). Prediction analyses employed repeated 10-fold cross-validation implemented using the scikit-learn toolbox. For each repetition, participants were randomly divided into training and testing sets using shuffled K-fold partitioning. Within each training fold, neural features and symptom variables were independently standardized using z-score normalization, and scaling parameters estimated from the training data were subsequently applied to the corresponding testing data to avoid information leakage across folds. Linear regression models were trained on the training set and subsequently used to predict symptom scores in the held-out testing set. Predicted symptom values were transformed back into the original scale using inverse normalization procedures. This process was repeated across five independent cross-validation repetitions with different random initialization seeds to improve robustness and reduce sampling-related variability. Prediction performance was quantified using Pearson correlation coefficients between predicted and observed symptom values, as well as mean squared error (MSE). Prediction analyses were performed separately within personality-defined subgroups to characterize subgroup-specific brain–symptom coupling during fear learning.

### Behavioral Analysis

Subjective fear ratings were collected before and after the fear conditioning procedure using a 10-point scale ranging from 1 (low fear) to 9 (extreme fear). Behavioral fear-learning responses were quantified as the change in fear ratings from pre- to post-conditioning. Because two conditioned threat cues (CS+1 and CS+2) were included in the paradigm, behavioral responses were averaged across the two CS+ conditions. Fear-learning effects were assessed by comparing changes in fear ratings between conditioned threat cues (CS+) and safety cues (CS−). Group differences between personality-defined subgroups were evaluated using two-sample t-tests.

### Statistical Analysis

Statistical analyses were performed using Python-based scientific computing libraries, including SciPy, scikit-learn, statsmodels, and Nilearn. Between-group differences in behavioral measures, symptom scores, neural decoding performance, task-related activation, and dynamic functional connectivity were evaluated using two-sample t-tests. Effect sizes were quantified using Cohen’s d to characterize the magnitude of subgroup differences. To minimize potential confounding effects associated with multi-site data acquisition, site-related variability in neuroimaging measures was harmonized using CovBat harmonization procedures while preserving biologically relevant variance associated with age, site and sex^68^. Brain activation differences were further assessed using second-level general linear models in Nilearn with non-parametric permutation testing (10,000 permutations) and Threshold-Free Cluster Enhancement (TFCE), with family-wise error correction applied across the whole brain (p_FWE < 0.05). Brain–symptom predictive analyses were performed using repeated cross-validated linear regression models. Prediction performance was quantified using Pearson correlation coefficients between predicted and observed symptom scores, as well as mean squared error (MSE). Correlational analyses were performed using Pearson correlation coefficients. Unless otherwise specified, statistical significance was defined as two-tailed p < 0.05.

## Supplementary Material

Supplementary Files

This is a list of supplementary files associated with this preprint. Click to download.

- Supplement.docx

## Figures and Tables

**Figure 3.**
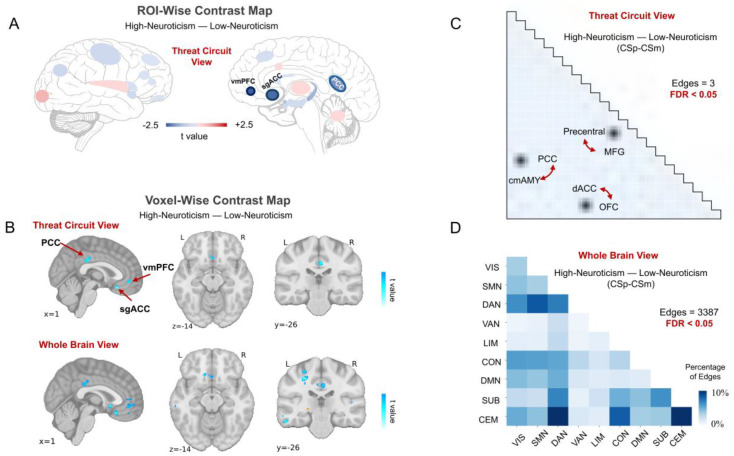
Personality-defined differences in fear-learning-related activation and dynamic functional connectivity. A. ROI-wise contrast maps showing subgroup differences in fear-learning-related activation (CSp−CSm) within the 24-node threat-circuit atlas. B. Voxel-wise contrast maps showing stronger activation in the low-neuroticism subgroup compared with the high-neuroticism subgroup during early fear learning, including vmPFC, sgACC, and PCC regions (p_FWE < 0.05). C. Dynamic functional connectivity differences within the threat-circuit atlas during fear learning (CSp−CSm). Significant subgroup differences were observed for PCC–cmAMY, dACC–OFC, and precentral–MFG connections (p_FDR < 0.05). D. Whole-brain network-level dynamic functional connectivity differences during fear learning. Significant subgroup differences were observed across DAN–CEM, CEM–CEM, CON–CEM, and DAN–SMN network interactions (p_FDR < 0.05). Overall, the low-neuroticism subgroup exhibited stronger fear-learning-related activation and connectivity compared with the high-neuroticism subgroup. For visualization purposes, activation and connectivity maps are displayed using the contrast High-Neuroticism − Low-Neuroticism; therefore, negative values (blue) indicate stronger fear-learning-related activation or connectivity in the low-neuroticism subgroup.

## Data Availability

The code used for personality clustering, neural decoder analysis, task-evoked activation analysis, dynamic functional connectivity (Jackknife), and brain–symptom prediction analyses is publicly available at: https://github.com/kaizhang0912/FearConditioning_Personality/tree/main. Analyses were implemented using Python and open-source scientific computing libraries, including Nilearn for fMRI processing: https://nilearn.github.io/stable/index.html, scikit-learn for machine learning analyses, statsmodels for statistical modeling, CovBat for harmonization: https://github.com/andy1764/CovBat_Harmonization, and Seaborn: https://seaborn.pydata.org/ for visualization.

## Data availability statement

The data supporting the findings of this study are available from the corresponding author upon reasonable request. Data sharing is subject to institutional review and must comply with institutional regulations and NIH data-sharing policies to ensure the protection of participant privacy.
